# Network pharmacology and molecular docking approach to elucidate the mechanisms of Liuwei Dihuang pill in diabetic osteoporosis

**DOI:** 10.1186/s13018-022-03194-2

**Published:** 2022-06-14

**Authors:** Zhaoqi Lu, Minling Huang, Haixiong Lin, Gaoxiang Wang, Huilin Li

**Affiliations:** 1 The Fourth Clinical Medical College, Guangzhou University of Chinese Medicine, Guangzhou, Guangdong China; 2 Department of Diabetes, Central Clinical School, Monash University, Melbourne, VIC Australia; 3 Ningxia Hui Autonomous Region Hospital and Research Institute of Traditional Chinese Medicine, Yinchuan, Ningxia China; 4 Institute for Tissue Engineering and Regenerative Medicine, The Chinese University of Hong Kong, Shatin, Hong Kong SAR China; 5 The First School of Clinical Medicine, Guangzhou University of Chinese Medicine, Guangzhou, China; 6 Shenzhen Traditional Chinese Medicine Hospital Affiliated to Nanjing University of Chinese Medicine, Shenzhen, Guangdong China; 7 Department of Endocrinology, Shenzhen Traditional Chinese Medicine Hospital, Shenzhen, Guangdong China

**Keywords:** Liuwei Dihuang pill, Diabetic osteoporosis, Network pharmacology, Molecular mechanism, Bioinformatics analysis, Pharmaceutical discovery

## Abstract

**Background:**

Diabetic osteoporosis (DOP) is one of the chronic complications of diabetes mellitus, but without a standardized treatment plan till now. Liuwei Dihuang pill (LDP) has gradually exerted a remarkable effect on DOP in recent years; its specific mechanism is not clear yet.

**Methods:**

We adopted network pharmacology approaches, including multi-database search, pharmacokinetic screening, network construction analysis, gene ontology enrichment analysis, Kyoto Encyclopedia of Genes and Genomes pathway analysis and molecular docking to elaborate the active components, signaling pathways and potential mechanisms of LDP in the treatment of DOP.

**Results:**

Twenty-seven active ingredients and 55 related disease targets have been found through integrated network pharmacology. Functional enrichment analysis shows that five key active ingredients, including beta-sitosterol, stigmasterol, diosgenin, tetrahydroalstonine, and kadsurenone, may give full scope to insulin secretion estrogen-level raising and angiogenesis in biological process through the pivotal targets. In addition, the underlying effect of PI3K/AKT/FOXO and VEGF pathways is also suggested in the treatment.

**Conclusion:**

Based on systematic network pharmacology methods, we predicted the basic pharmacological effects and potential mechanisms of LDP in the treatment of DOP, revealing that LDP may treat DOP through multiple targets and multiple signaling pathways, which provide evidence for the further study of pharmacological mechanism and broader clinical thinking.

**Supplementary Information:**

The online version contains supplementary material available at 10.1186/s13018-022-03194-2.

## Introduction

Along with the significant change in an individual’s lifestyle and diet habits, diabetes mellitus (DM) has gradually developed into a common endocrine and metabolic disease that accompanies a dramatically increasing prevalence worldwide. Osteoporosis (OS) is also a growing public health concern worldwide relating to the possibility of a loss of life, with more than 9 million osteoporotic fractures taking place every year [1]. More and more clinical studies have pointed out the close relationship between glucose levels in the body and bone metabolism indicates that DM may increase the risk of OS and a series of skeletal system complications. Epidemiological surveys lately demonstrated that the risk of OS in DM patients is 4–5 times of non-DM patients [2]. DOP is a chronic complication of DM in the skeletal system, mainly manifested as low bone mass, increased bone fragility, decreased bone quality, and increased fracture risk. DM may induce a series of metabolic disorders, such as inflammation and oxidative stress [3]. Meanwhile, it could lead to the imbalance of hormone levels, a disorder of calcium and phosphorus metabolism, which may ultimately arouse the dynamic imbalance of bone construction remodeling and changes in bone microscopic and functional structure.

Liuwei Dihuang pill (DOP) is a classic prescription for nourishing kidney yin created by Qian Yi in the Song Dynasty. It consists of six traditional Chinese medicines: Rehmanniae Radix Preparata (SDH), Cornus Officinalis Sieb.et Zucc. (SZY), Poria cocos (Schw.) Wolf. (FL), Rhizoma Dioscoreae (SY), Alisma orientale (Sam.) Juz. (ZX), and Cortex Moutan (MDP). In traditional Chinese medicine, it is believed [4] that “kidney governs the bones and can breed bone marrow,” and the “tonifying the kidney therapy” in Chinese medicine can be used to treat bone atrophy. Modern clinical data have effectively confirmed that LDP is a safe and effective prescription, which has significant effects on both DM and OS [5], and can profoundly improve the glucose and lipid metabolism and osteocalcin (OC) levels in patients with DOP [6].

**Fig. 1:**
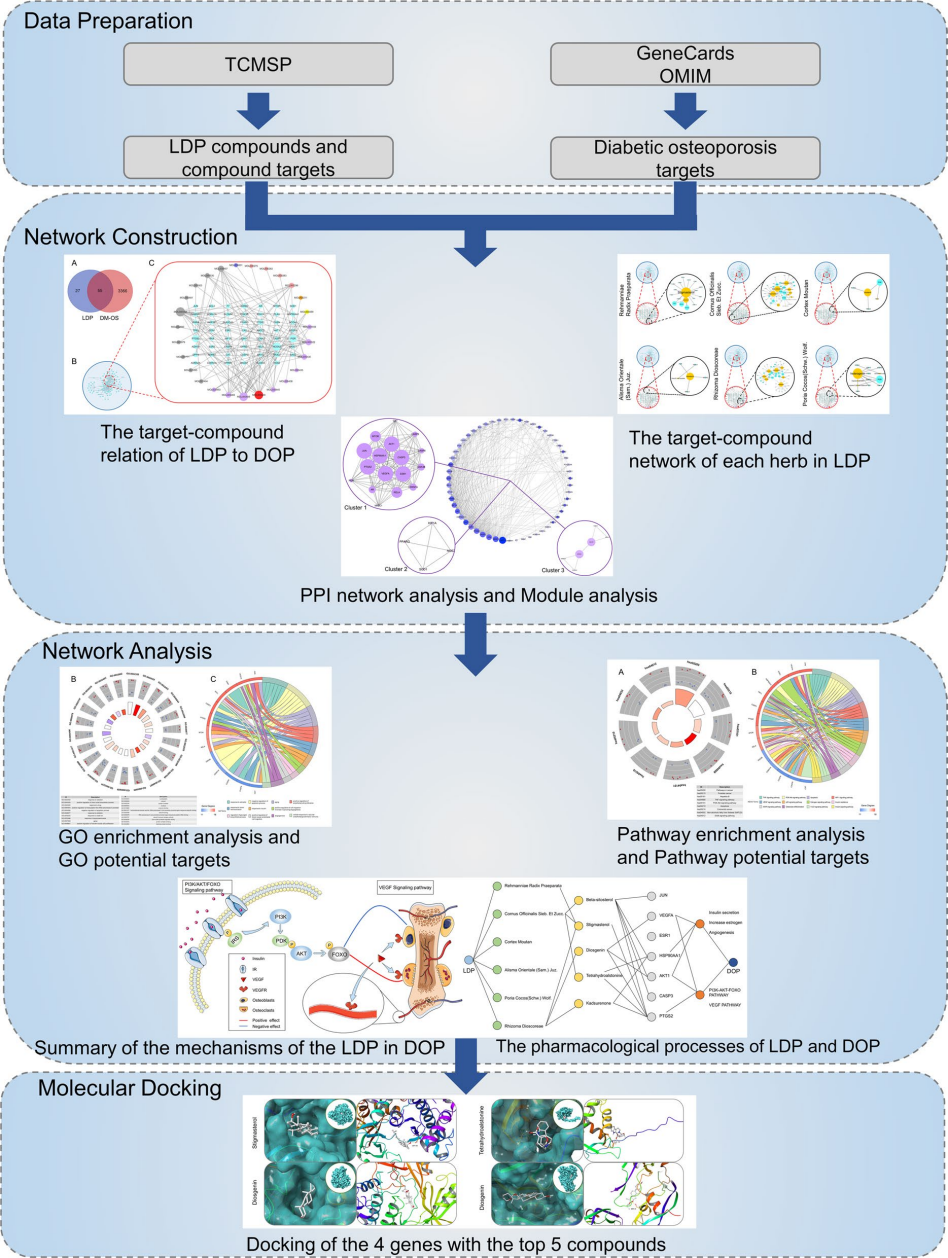
Workflow for LDP in the treatment of DOP

Network pharmacology is an emerging discipline with scientific ideas and research strategies. It provides a new network model of “multiple targets, multiple effects, and complex diseases” to explain the mechanism of traditional Chinese medicine treatment [7]. At present, the clinical efficacy of LDP has been proven, but its complex pharmacological effects and mechanism of action are still unclear. This study aims to use the network pharmacology and molecular docking methods to investigate target genes, signaling pathways, and molecular mechanisms to expand further great ideas for DOP clinical treatment and related scientific research fields. Figure 1 shows the flowchart of the network pharmacology study on LDP in DOP treatment.

## Methods

### Herbal compounds and targets in LDP

Active components of the six LDP herbs were obtained from the Traditional Chinese Medicine Systems Pharmacology Database and Analysis Platform (TCMSP, http://lsp.nwu.edu.cn/, Version 2.3) [8]. In this study, oral bioavailability (OB) ≥ 30%, drug-like (DL) ≥ 0.18, and Caco-2 permeability (Caco-2) ≥ 0.4 must be met at the same time. Previous studies have shown that compounds with OB ≥ 30% have higher oral absorption and slow metabolism [9], while compounds with DL ≥ 0.18 have better effects in drug development [10]. Caco-2 can predict the intestinal absorption rate and the oral dose of ingredients in the human body. Since Caco-2 < 0.4 is impermeable, the threshold of Caco-2 permeability is set to 0.4 [11].

Subsequently, the TCMSP was further used to predict the potential targets of these bioactive compounds. We converted the obtained drug targets into gene names and restricted the species to “Homo sapiens” through The Universal Protein Resource (UniProt, http://www.uniprot.org/) [12].

### Diabetic osteoporosis targets

In this study, human genes related to LDP came from two sources: (1) GeneCards (https://www.genecards.org/) database [13]. It is a comprehensive database that provides comprehensive information about all annotations and predictions of human genes, including genome, transcriptome, proteomics, genetic, clinical, and functional information. (2) OMIM database (http://www.omim.org/) [14] is a comprehensive and authoritative collection of human genes, including more than 15,500 genes, 26,200 allele variants, and 7,800 genetic phenotypes. Through the search term “diabetic osteoporosis,” we obtained 3421 items for analysis after removing duplicate genes. The intersection of DOP and LDP drug targets (D&L) is considered to be the potential genes for LDP to treat DOP.

The compound–D&L target interaction was established and visualized by Cytoscape (Version 3.7.1). By using the Cytoscape tool network analyzer to calculate three indicators: degree, intermediateness, and compactness, we evaluated the topological characteristics of the interactive network. We also adopted the MCC algorithm of another Cytoscape tool—cytoHubba [15]—to perform further precise analysis of compounds with multiple target connections in the network. These compounds are considered the core compounds of the network and play an important role in the treatment of DOP.

### Protein–protein interaction data

D&L's protein interaction (PPI) data are obtained from Search Tool for the Retrieval of Interacting Genes (STRING, https://string-db.org/), which contains 9,643,763 proteins and 1,380,838,440 protein interaction information [16]. We restricted the search condition to “Homo sapiens” and PPI with a comprehensive score higher than 0.4 for further research. In addition, the PPI network was built up in Cytoscape. We calculated topological characteristics of the interaction network and adjusted the size and color of the nodes according to the degree and intermediateness. Finally, we used Cytoscape's plug-in MCODE [17] to obtain the correlation among network clusters and picked out the hub genes with high connectivity. Degree cutoff = 2, Node Score Cutoff = 0.2, and K-Core = 2 were selected as the optimal options [18].

### GO enrichment analysis and KEGG pathway analysis

Gene ontology (GO) functions are performed in three categories of GO terms: biological process (BP), molecular function (MF), and cellular component (CC). The “Kyoto Encyclopedia of Genes and Genomes” (KEGG) pathway enrichment analysis is an important method for characterizing candidate targets. Both of them were conducted to predict the specific mechanism through the Database for Annotation, Visualization, and Integrated Discovery (DAVID, https://david.ncifcrf.gov/). We set the standard of p value to 0.05 and use RStudio 3.6.1 (GOplot) to visualize GO terms and KEGG pathways, and the false discovery rate (FDR) is < 0.05.

### Validation of compound–target Interaction

We chose the core compounds in LDP and the key proteins in core pathways for molecular docking. First, the 3D structure of the target protein was obtained from the RCSB PDB database (https://www.rcsb.org/) and PubChem (https://pubchem.ncbi.nlm.nih.gov/) was used to find the chemical and conformational information of the compounds. Then, before the docking experiments, we used AutoTools software to preprocess the crystal structure of the target protein, including removing excess protein chains, ligands, water molecules, and hydrogenation. Moreover, the ligand structure is needed to conform to a low-energy conformation. Furthermore, we used AutoDock Vina to simulate the docking state between proteins and small molecules [19]. Finally, we used Schrodinger software to analyze its preferred conformation and draw simulation diagrams.

## Results

### Compounds and targets of LDP

**Fig. 2:**
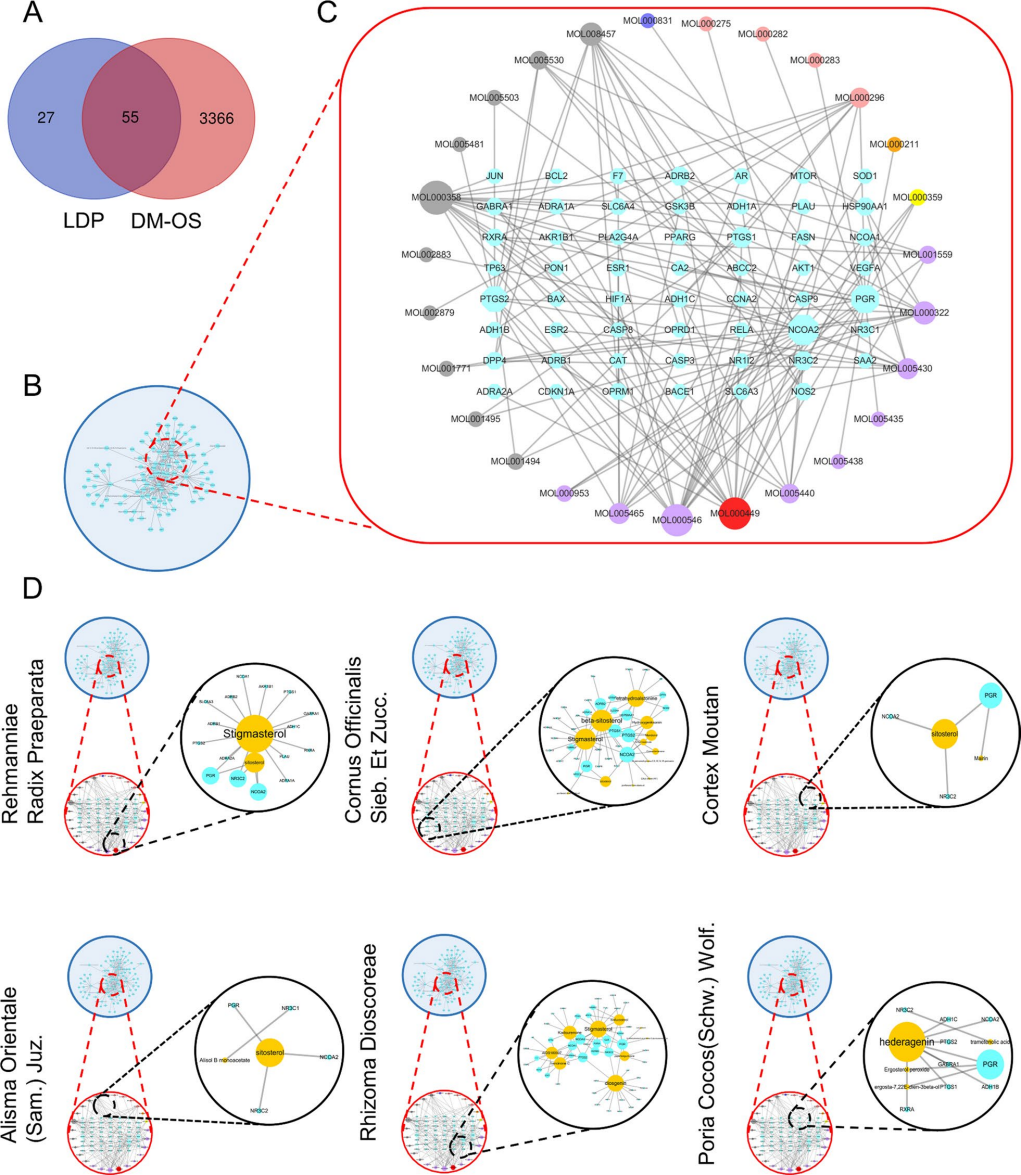
Composite target relationship between LDP and D&L. (**a**) The quantity of intersecting LDP and DOP target genes. (**b**) LDP's composite target network. (**c**) The compound target network based on D&L intersection. The gene targets are described as sky blue hexagons. The purple, gray, orange, pink, and blue nodes represent SY, SZY, MDP, FL, and ZX, respectively. The red node represents common compounds of SDH, SZY, and SY. The yellow node represents the common compound of SDH, SZY, MDP, and ZX. The nodule size of gene targets and compounds is directly proportional to the degree. The line represents the relationship between the compound and target node. (**d**) Compound target network of each herb in LDP. The blue round frame represents the composite target network of LDP, and the red round frame represents the combined target network of D&L. The black round frame represents each herb's compound target network in LDP, the sky-blue node represents the target, and the yellow node represents the corresponding compounds

According to the three conditions of OB ≥ 30%, DL ≥ 0.18, and CaCO-2 ≥ 0.4, we screened a total of 28 compounds and 82 gene targets of LDP on the TCMSP platform after converting the acquired drug targets into gene names and removing the duplicates (Fig. 2b). We have discovered that some unique compounds interact with multiple targets and participate in the supervision of multiple targets. This indicates that these compounds may have complementary and synergistic therapeutic effects in treating this disease. For example, it is 81 in stigmasterol, 28 in beta-sitosterol, 25 in tetrahydroalstonine, 23 in kadsurenone, 18 in hancinone C, 17 in hederagenin, and 16 in diosgenin.

### The compound target network based on the D&L intersection

The 82 LDP compound targets obtained above were intersected with 3421 DOPs to obtain 55 LDP and DOP (D&L) genes (Fig. 2a). There are 82 nodes and 140 edges, of which there are 55 D&L target nodes and 27 compound nodes. Each edge represents the interaction between the compound target and the compound target, forming a compound D&L target network to illustrate the mechanism of LDP in treating DOP (Fig. 2c). Table 1 shows 27 kinds of compound information. The degree of a node indicates the number of routes required to connect to other nodes in the network. In this network, on average, each compound is linked to 5.19 targets and each target is connected to 2.55 compounds. A single compound can act on multiple targets, and multiple compounds can act on the same target. This phenomenon reflects the multi-component and multi-target characteristics of traditional Chinese medicine treatment. According to the topological properties and MCC algorithm, 18.5% of compounds could simultaneously connect to more than ten gene targets, indicating that these compounds can regulate most D&L targets. Thus, these compounds may be the key compounds in the network, and the top five ones are beta-sitosterol, stigmasterol, diosgenin, tetrahydroalstonine, and kadsurenone.

**Table 1:** Compound–compound target network information

MOL ID	Compound	Drug	OB (%)	DL	Caco-2	Degree
MOL000358	Beta-sitosterol	SZY	36.91	0.75	1.32	28
MOL000449	Stigmasterol	SDH/SZY/SY	43.83	0.76	1.44	27
MOL008457	Tetrahydroalstonine	SZY	32.42	0.81	0.90	25
MOL000322	Kadsurenone	SY	54.72	0.38	0.82	23
MOL005430	Hancinone C	SY	59.05	0.39	0.74	18
MOL000296	Hederagenin	FL	36.91	0.75	1.32	17
MOL000546	Diosgenin	SY	80.88	0.81	0.82	16
MOL005465	AIDS180907	SY	45.33	0.77	0.73	10
MOL001559	Piperlonguminine	SY	30.71	0.18	0.95	9
MOL005440	Isofucosterol	SY	43.78	0.76	1.36	9
MOL005530	Hydroxygenkwanin	SZY	36.47	0.27	0.52	8
MOL000359	Sitosterol	MDP/SZY/SDH/ZX	36.91	0.75	1.32	3
MOL000953	CLR	SY	37.87	0.68	1.43	3
MOL001494	Mandenol	SZY	42.00	0.19	1.46	3
MOL002879	Diop	SZY	43.59	0.39	0.79	3
MOL005503	Cornudentanone	SZY	39.66	0.33	0.47	3
MOL001495	Ethyl linolenate	SZY	46.10	0.20	1.54	2
MOL001771	Poriferast-5-en-3beta-ol	SZY	36.91	0.75	1.45	2
MOL000275	Trametenolic acid	FL	38.71	0.80	0.52	1
MOL000282	Ergosta-7,22E-dien-3beta-ol	FL	43.51	0.72	1.32	1
MOL000283	Ergosterol peroxide	FL	40.36	0.81	0.84	1
MOL000211	Mairin	MDP	55.38	0.78	0.73	1
MOL005435	24-Methylcholest-5-enyl-3belta-O-gluCopyranoside_qt	SY	37.58	0.72	1.33	1
MOL005438	Campesterol	SY	37.58	0.71	1.34	1
MOL002883	Ethyl oleate (NF)	SZY	32.4	0.19	1.4	1
MOL005481	2,6,10,14,18-Pentamethylicosa-2,6,10,14,18-pentaene	SZY	33.4	0.24	1.94	1
MOL000831	Alisol B monoacetate	ZX	35.58	0.81	0.46	1

### Compound target network of each herb in LDP

LDP is composed of Rehmanniae Radix Preparata (SDH), Cornus Officinalis Sieb.et Zucc. (SZY), Poria cocos (Schw.) Wolf. (FL), Rhizoma Dioscoreae (SY), Alisma orientale (Sam.) Juz. (ZX), and Cortex Moutan (MDP). Among the D&L genes, 16, 31, 9, 45, 4, and 3 genes are relatively corresponding to 2, 13, 4, 10, and 2 compounds. Cornus Officinalis Sieb. Et Zucc and Rhizoma Dioscoreae are the two herbs that account for the highest proportion of collected compounds and targets (Fig. 2d).

### PPI network of D&L targets and module analysis

**Fig. 3:**
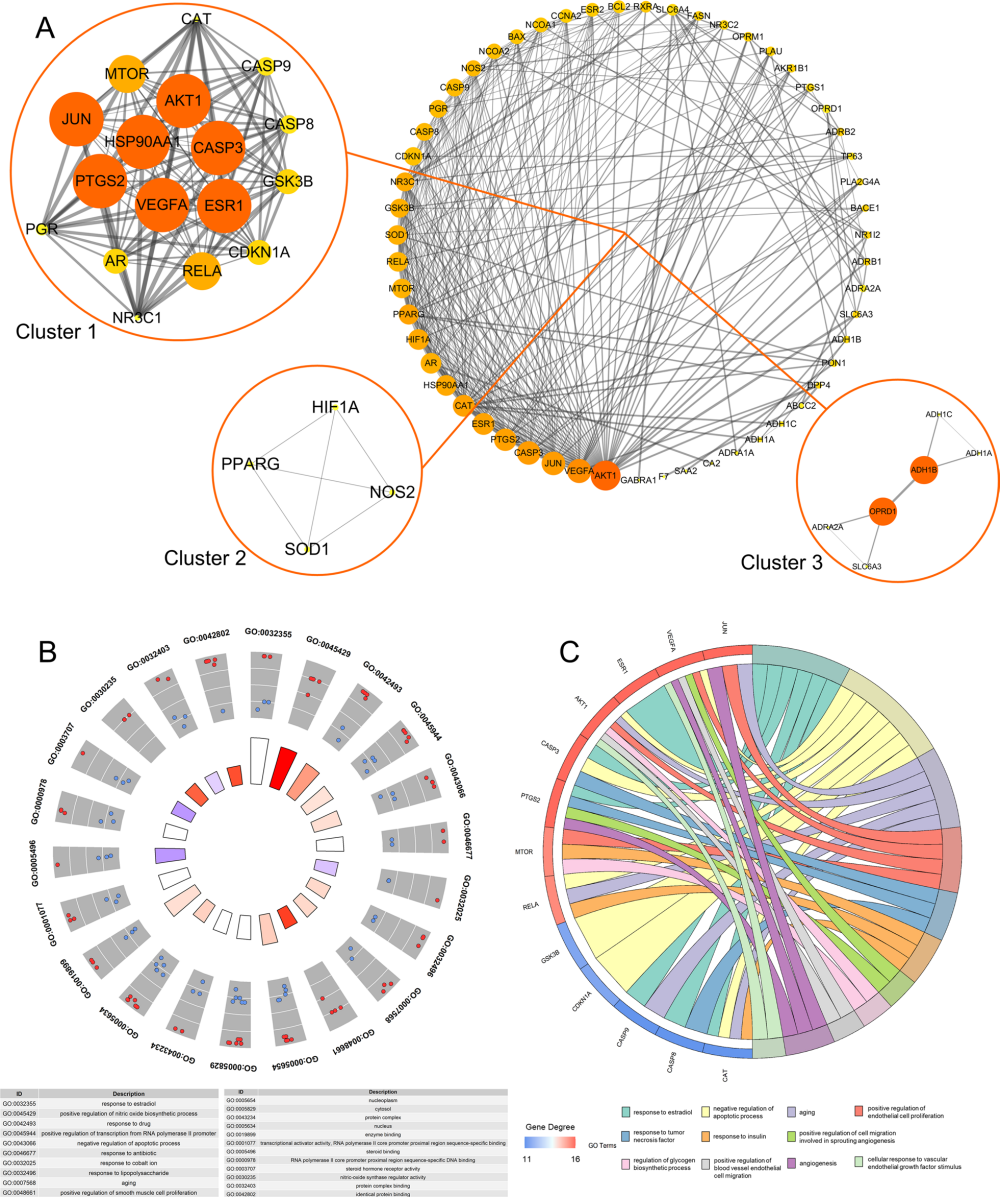
PPI and GO enrichment analysis. (**a**) D&L's PPI network. The size of the point represents the degree, the color depth represents the compactness, and the thickness of the line represents the intermediateness. (**b**) GO enrichment analysis of D&L (FDR < 0.05). The red node indicates that the degree is greater than or equal to 15, and the blue node indicates less than 15. The sum of the red and blue nodes represents the number of genes enriched in the corresponding terms. The height of the column in the inner circle is inversely proportional to the value of P. (**c**) Analysis of BP items closely related to LDP in DOP treatment

Fifty-five D&L genes were imported into the STRING database (version: 11.0) for protein–protein interaction analysis. As shown in Fig. 3a, a total of 332 pairs of interactions were obtained. In this network, the average connection degree of each node is 12.1. The higher the degree of the node, the more influential the node is. Thus, the degree of the following nodes is greater than 20: for AKT1, it is 38. Vascular endothelial growth factor A(VEGFA) is 28; CASP3 is 27; June 27; PTGS2 is 25; ESR1 and CAT are 24; for AR, HSP90AA1 and HIF1A, it is 22; PPARG is 21; MTOR, SOD1, and RELA are 20. We clustered the targets in the PPI network and obtained three modules (Fig. 3a). The clustering score of each module represents the core density of the nodes and the adjacent nodes in the topology, indicating that the more concentrated the core, the more concentrated the cluster. This result shows that LDP can play a synergistic role in DOP treatment through these three aspects. Module 1 has 17 targets, 122 interactions, and a score of 15.25; Module 2 has four targets, six interactions, and a score of 4; Module 3 has six targets, seven interactions, and a score of 2.8. Further topology analysis of the Module 1 network reveals that the seven most advanced targets are JUN, VEGFA, ESR1, HSP90AA1, AKT1, CASP3, and PTGS2. These targets are considered the hub nodes in module 1 and core targets in the PPI network, so we speculate that they may play a key role in DOP treatment.

### GO enrichment analysis of targets in module 1

To further clarify the core biological functions of LDP, GO analysis was performed on 17 potential targets in Module 1 from the aspects of biological process (BP), molecular function (MF), and cell composition (CC).

In the case of *P* < 0.05, we obtained 133 GO entries, including 90 BP entries, 31 MF entries, and 12 CC entries (Additional file 1: Table S1). At the same time, we found that 11 BP entries were significantly related to DOP (Fig. 3c): response to estradiol; negative regulation of apoptotic process; aging; positive regulation of endothelial cell proliferation; response to tumor necrosis factor; response to insulin; positive regulation of cell migration involving sprouting angiogenesis; regulation of glycogen biosynthetic process; positive regulation of vascular endothelial cells migration; angiogenesis and cellular response to vascular endothelial growth factor stimulation. At the same time, according to FDR < 0.05, we clearly know that there are 22 items, of which ten items are in BP, eight items in MF, and four items in CC (Fig. 3b).

### KEGG enrichment analysis of targets in Module 1

**Fig. 4:**
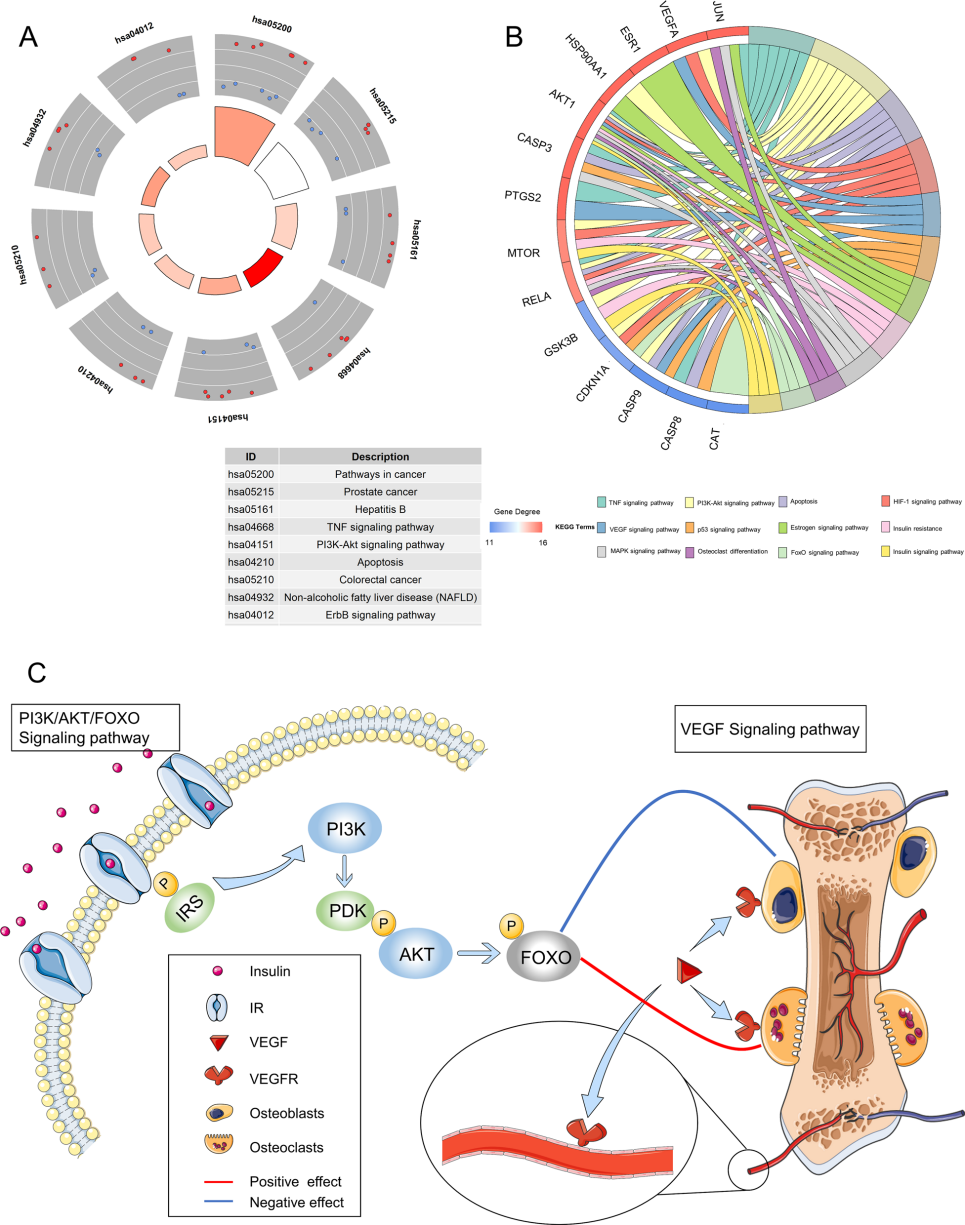
Schematic diagram of mechanism in DOP. (**a**) KEGG enrichment analysis (FDR < 0.05). (**b**) Analysis of the KEGG pathway closely related to LDP in DOP treatment. **(c**) The mechanism of PI3K/AKT/FOXO signaling pathway and VEGF signaling pathway in DOP

In module 1, according to *P* < 0.05 (Additional file 1: Table S2), a total of 56 signal pathways were significantly enriched, while FDR < 0.05 identified nine signal pathways: prostate cancer; colorectal cancer; TNF signaling pathway; PI3K-Akt signaling pathway; apoptosis ErbB signaling pathway; hepatitis B; and non-alcoholic fatty liver (Fig. 4a).

Twelve signal pathways with a *P* value of less than 0.05 are significantly related to DOP (Fig. 4b), which mainly include TNF signaling pathway, PI3K-Akt signaling pathway, apoptosis, HIF-1 signaling pathway, VEGF signaling pathway, p53 signaling pathway, estrogen signaling pathway, insulin resistance, MAPK signaling pathway, osteoclast differentiation, the forkhead box O (FOXO) signaling pathway, and insulin signaling pathway. We further explored and found that the interaction between the above pathways may be a potential mechanism for LDP to treat DOP. On the one hand, LDP activates the downstream PI3K/AKT signaling pathway through the insulin signaling pathway and mediates the phosphorylation of FOXO, resulting in hypoglycemic and osteogenic effects. On the other hand, the cascade of diabetes–angiopathy–bone destruction indicates that blood vessels are closely related to the pathogenesis of DOP. Therefore, the VEGF signaling pathway is considered one of the core pathways of LDP (Fig. 4c).

### Molecular docking verification

In order to further explore the interactions between the top five compounds of LDP and related genes in the core pathway and to clarify a new drug development strategy, further molecular docking simulations were carried out. Through docking simulation, we identified the binding affinity between the top five compounds (beta-sitosterol, stigmasterol, diosgenin, tetrahydroalstonine, and kadsurenone) and the core target protein (AKTI, FOXO1, PI3K, and VEGFA) in PI3k/Akt signaling pathway and VEGF signaling pathway. After searching AKTI, FOXO1, PI3K, and VEGFA from the PDB protein database, the three-dimensional structures of 6s9w, 6qvw, 3l54, and 3qtk proteins were obtained.

**Fig. 5:**
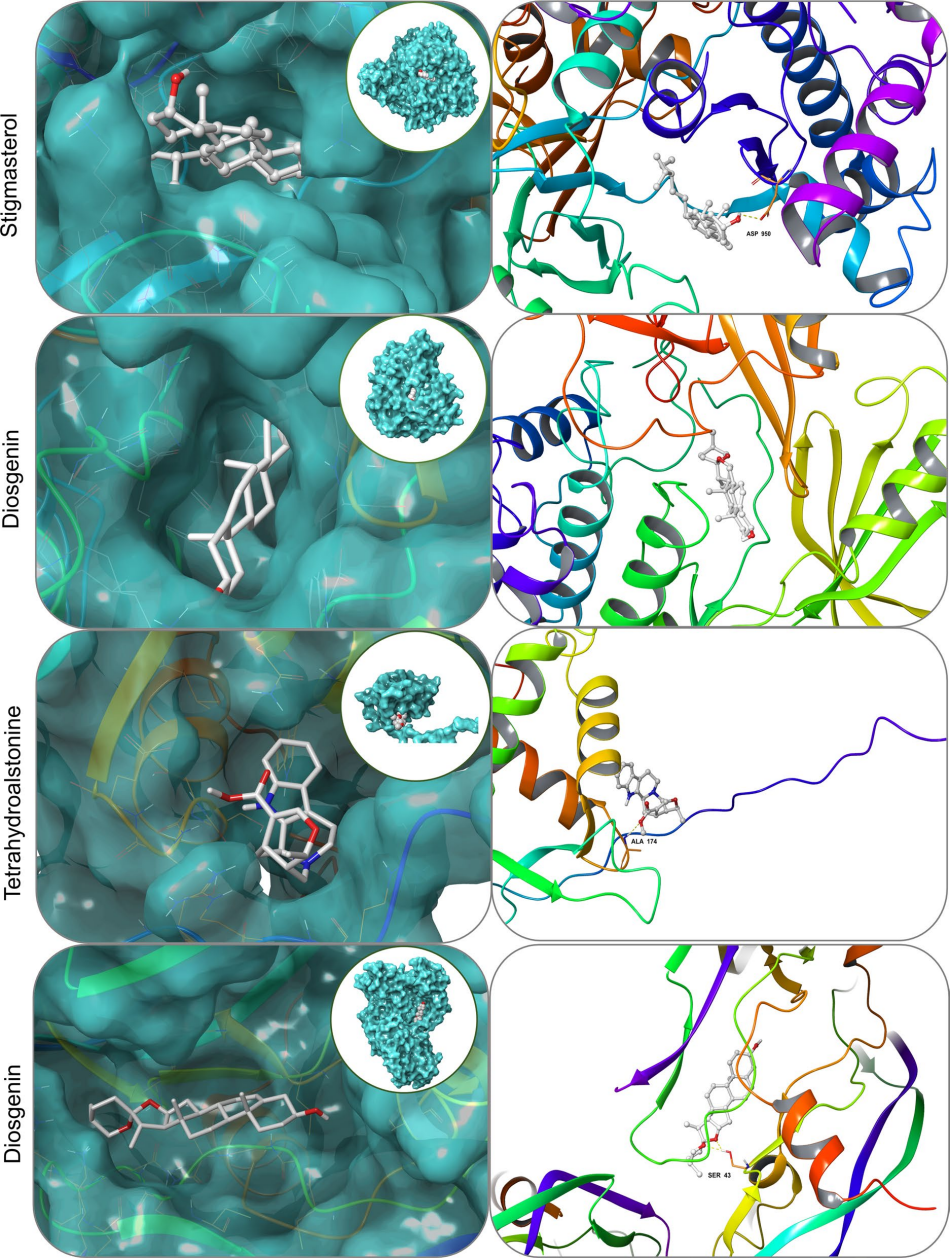
Four best docking results of five compounds and the core targets. The docking between stigmasterol and PI3K, diosgenin and AKT1, tetrahydroalstonine and FOXO1, diosgenin and VEGFA

The docking score suggests that most of them have excellent binding activity (Table 2), which indicates that LDP may affect DOP through PI3k/Akt signaling pathway and VEGF signaling pathway. Figure 5 shows the docking results of the compounds with the best binding affinity for each core target protein. Each small molecule can enter the active pocket of the protein, showing proper matching characteristics. Diosgenin and the SER 43 residue on the receptor protein VEGFA form two hydrogen bonds, which can stabilize the diosgenin–VEGFA complex. Diosgenin and AKT1 have the best binding activity, but a stable structure has not yet been established. The H bond can stabilize the tetrahydroalstonine–FOXO1 complex with residue ALA 174, and tetrahydroalstonine can tightly bind to the active pocket of FOXO1. H-bonds stabilize the stigmasterol–PI3K complex with residue ASP 950.

**Table 2:** Docking score of compounds with each core target

Compound	Structure	Docking score/(kcal mol^−1^)			
		AKT1	FOXO1	PI3K	VEGFA
Beta-sitosterol		− 11.7	− 6.5	− 9.0	− 7.2
Stigmasterol		− 11.9	− 6.7	− 9.3	− 7.6
Diosgenin		− 13.3	− 7.5	− 8.2	− 9.2
Tetrahydroalstonine		− 9.1	− 7.6	− 9.0	− 8.3
Kadsurenone		− 9.5	− 6.6	− 8.0	− 7.4

### Toxicity profiling

In the long-term clinical application and clinical trials, no liver and kidney toxicity and adverse reactions to Liuwei Dihuang pill were observed [6, 20]. Previous studies show that the central nervous system, respiratory system, and cardiovascular system of mice and rats were normal after oral administration of Liuwei Dihuang 15 g·kg^–1^, while there was no death in mice and rats after 14 days of administration of 15 g·kg^–1^ Liuwei Dihuang either [21]. Results of a study on the long-term toxicity of Liuwei Dihuang pill showed that the nontoxic dosage of oral and repeat administration was 0.42 g kg^–1^ in rats for 180 days and 0.5 g kg^–1^ in beagle dogs for 270 days, which is the equivalent of 25 and 30 times of the clinical dosage for humans, respectively [21]. Specifically, we studied the toxicity analysis of the top five compounds in Liuwei Dihuang pill on ADMETlab 2.0 (https://admetmesh.scbdd.com/) [22]. ADMETlab 2.0 is a powerful tool for the systematical evaluation of ADMET (absorption, distribution, metabolism, excretion, and toxicity) properties, as well as some physicochemical properties and medicinal chemistry friendliness [23]. According to the reports on the Web site, beta-sitosterol, stigmasterol, diosgenin, tetrahydroalstonine, and kadsurenone have no obvious toxicity to the liver, heart, respiration, skin, eyes, etc., and no apparent toxicity was observed in the oral toxicity test in rats (Table 3). All the above have indicated the high safety of Liuwei Dihuang pill.

**Table 3:** Toxicity profiling of core compounds

Property	Range reference	Value				
		Beta-sitosterol	Stigmasterol	Diosgenin	Tetrahydroalstonine	Kadsurenone
hERG blockers	1: active 0: inactive	0.049	0.012	0.029	0.911	0.055
Human hepatotoxicity	1: positive 0: negative	0.16	0.011	0.204	0.6	0.42
Drug-induced liver injury	1: high risk 0: no risk	0.203	0.055	0.057	0.07	0.09
AMES toxicity	1: positive 0: negative	0.026	0.029	0.053	0.754	0.018
Rat oral acute toxicity	1: high 0: low	0.018	0.054	0.748	0.957	0.235
Maximum recommended daily dose	1: FDAMDD (+) 0: FDAMDD (−)	0.73	0.539	0.646	0.959	0.826
Skin sensitization	1: sensitizer 0: non-sensitizer	0.133	0.025	0.241	0.098	0.211
Carcinogen city	1: carcinogen; 0: non-carcinogen	0.047	0.054	0.188	0.972	0.887
Eye corrosion	1: corrosive 0: noncorrosive	0.003	0.003	0.003	0.003	0.003
Eye irritation	1: irritant 0: nonirritant	0.01	0.01	0.009	0.013	0.012
Respiratory toxicity	1: toxicant; 0: nontoxicant	0.536	0.19	0.517	0.976	0.958

### Dynamic behavior of drug under controlled conditions

Although Liuwei Dihuang pill has a long history of clinical application, the scientific and systematic pharmacokinetic evaluation is far from comprehensive. There are few studies on the pharmacokinetic parameters and pharmacokinetic characteristics of Liuwei Dihuang pill, which mainly focus on its bioactive chemical components. The effects of Liuwei Dihuang pills on cytochrome P4502C19, cytochrome P4502D6, and cytochrome P4503A4 activities in 12 healthy Chinese subjects were evaluated in a single-center, controlled, open-label, two-way crossover clinical trial [24]. The results showed that there was no significant difference in the activities of the three tested enzymes before and after 14 days of administration of Liuwei Dihuang pill, which had no effect on the pharmacokinetic parameters of the substrates and the metabolites and did not affect the activities of CYP2C19, CYP2D6, and CYP3A4 [24]. However, it induced cytochrome P4501A2 (CYP1A2), inhibited the activity of cytochrome P4502A6 (CYP2A6) and NAT2, and affected human caffeine metabolism [25]. In another study, after the oral administration, nine compounds were identified in blood as constituents derived from the pill: 5-hydroxymethyl-2-furoic acid (HMFA), morroniside, sweroside, loganin, paeonol, and paeoniflorin were the main constituents absorbed into the blood, whereas the others were the metabolites of paeonol [26]. The pharmacokinetic analysis indicated that the t_1/2α_ and t_1/2β_ were 2.62/32.66, 0.46/4.71, and 1.30/23.51 h, and the climax times and concentrations were 0.56/683.75, 0.70/2826.11, and 0.62 h/ 4030.48 ng·mL^−1^ for HMFA, loganin, and paeonol, respectively [26]. However, unfortunately, we have not further found the specific pharmacokinetic experimental study of Liuwei Dihuang pill for diabetic osteoporosis. In the future, we should pay more attention to the metabolism and transformation of drugs in the body as well as network and algorithm prediction.

## Discussion

Network pharmacology is a combination of clustering algorithms and network topology. It is good at expressing complex data interaction relationships. It is a huge project aimed at exploring the relationship between compounds and target genes of the disease through experiments. Therefore, we eliminate irrelevant interference factors through network pharmacology, aiming to screen the central compounds, the most potential gene targets, and pathways to provide a more precise direction for studying its mechanism.

### Central compounds analysis

Cornus Officinalis Sieb. et Zucc. and Rhizoma Dioscoreae are the two main drugs that can adjust most compounds and targets in the LDP component analysis. After further research on herbal medicine, we found that five central compounds (beta-sitosterol, stigmasterol, diosgenin, tetrahydroalstonine, and kadsurenone) play a critical regulatory role in the treatment of DOP. Molecular docking verification showed that these five compounds also have a good degree of binding with the core protein of the vital pathway we obtained (of which diosgenin is the most important). In vitro experiments have shown that diosgenin can reduce bone loss in ovariectomized rats by reducing the ratio of receptor activator of nuclear factor-κ B ligand (RANKL)/osteoprotegerin (OPG) [27], while in vivo experiments have shown that that it may also promote mouse embryonic osteoblast precursor cells (MC3T3-E1 cells) proliferation and stimulate the synthesis and secretion of type 1 collagen, alkaline phosphatase, and osteopontin [28]. At the same time, diosgenin also plays a significant role in bone formation by up-regulating VEGFA and activating angiogenesis [29]. In addition, previous studies have shown that diosgenin can enhance the insulin-dependent glucose uptake of MC3T3-L1 cells [30] and then affect the absorption and utilization of glucose by skeletal muscle through Akt phosphorylation [31]. The signaling pathway can regulate glucose metabolism through PI3K/Akt signaling pathway, improve insulin resistance, and play a key role in regulating blood sugar [32]. In the above work, we outlined a new type of compound that shows an unexpected effect in DOP treatment. Its mechanism on OS and DM has been cleared, but no relevant report on DOP has been found. Therefore, through this study, combined with molecular docking results, we believe that diosgenin is a compound with great potential in the treatment of DOP. It may occupy a promising position through PI3K/AKT/FOXO and VEGF signaling pathways, and it is worthy of further discussion and research.

### Potential targets analysis

To clarify the role of LDP in gene function, we performed GO function enrichment analysis on potential targets. The biological process shows that insulin secretion, increasing estrogen levels, and promoting angiogenesis are the three main aspects that LDP may play an important role in the treatment of DOP.

The lack of insulin (INS) is directly related to the occurrence and development of DOP. INS sends signals to the insulin receptor (IR) on the surface of osteoblasts (OBs) to promote collagen synthesis and calcium deposition in bones [33]. Osteocalcin (OC) is the main non-collagen protein formed from the inorganic matrix of bone. It is a specific marker of bone turnover and bone formation. The level of OC is positively correlated with the INS signal of OB: INS stimulates OB to increase the expression of OC; while eliminating the IR in the OB, INS signal transmission is prevented, and the OC expression is reduced accordingly [34]. In patients with poor blood sugar control, especially T1DM, OC transcription, and serum levels are reduced, but they can be partially recovered after treatment with INS [35]. Therefore, insufficient INS secretion and reduced IR can affect OB activity, OC levels, and bone formation.

Sclerostin is secreted by bone cells and is a negative regulator of bone mass, which can effectively prevent the bone formation and promote osteoblast apoptosis. Experiments have shown that the expression of the sclerostin gene is increased in diabetic rats [36], while bone cells treated with high glucose or advanced glycation end products (AGEs) show increased expression of sclerostin and mRNA, accompanied by bone cell apoptosis [37]. The above indicates that the high-glucose environment will lead to bone loss by up-regulating sclerostin expression. However, the secretion secreted by bone cells is regulated by estrogen, and increased estrogen will cause the level of sclerostin to decrease. In the experiment of ovariectomized SD rats, subcutaneous injection of estradiol can effectively reduce the expression of sclerostin, thereby increasing bone mass [38]. Therefore, stimulating the estradiol synthesis helps increase bone mass and improve DOP.

Bone is a connective tissue rich in blood vessels. As an influencing factor in bone development, regeneration, and remodeling, blood vessels are essential in reducing bone formation caused by impaired angiogenesis. Clinical studies have shown that decreased bone mineral density in women with Type 2 diabetes mellitus (T2DM) has a special relationship with diabetic microangiopathy [39]. In the streptozotocin (STZ)-induced DM mouse model, the number of femoral blood vessels was significantly reduced. At the same time, the expression of platelet endothelial cell adhesion molecule (CD31) and vascular endothelial growth factor (VEGF) is also reduced, and bone formation is reduced [40]. Promoting angiogenesis in T2DM mice can effectively improve bone formation and bone remodeling during long bone regeneration [41]. In short, LDP can interfere with DOP through the vascular osteogenesis coupling effect.

### Core pathways analysis

In addition, combined with the GO enrichment analysis and based on the enrichment results of KEGG, we speculate that the pathway of LDP on DOP may be mainly insulin-induced PI3K/AKT/FOXO signaling pathway and VEGF signaling pathway.

#### PI3K/AKT/FOXO signaling pathway

The insulin signaling pathway is of great significance for maintaining average blood glucose levels and avoiding complications of hyperglycemia. In the insulin pathway, insulin can activate multiple intracellular signaling pathways, among which PI3K/AKT is the main downstream molecular pathway downstream and plays a central role in cell physiology. PI3K/AKT signaling pathways can effectively improve insulin sensitivity, relieve insulin resistance, and regulate glucose metabolism [42]. It also participates in the key regulation of bone growth and bone remodeling, maintaining the balance between osteogenesis and bone destruction [43]. Previous experiments have confirmed that the activation of the PI3K/AKT signaling pathway may enhance the proliferation activity of osteoblasts (OB). Its inhibition effect will have a negative impact on the osteoblastic process of osteoblasts [44]. For example, AKT1 knockout mice have shorter bones, and AKT1 and AKT2 knockout mice have delayed ossification. At the same time, PI3K/AKT signaling pathway is also related to diabetes-related bone disease. In human bone marrow mesenchymal stem cells cultured with high glucose, activation of the PI3K/AKT pathway can attenuate the proliferation and differentiation of osteoclasts induced by high glucose [45–47].

The PI3K/AKT signaling pathway is a typical pathway to regulate the transcriptional activity of FOXO [48]. Through phosphorylation of FOXO in the downstream FOXO signaling pathway, the PI3K/AKT pathway is introduced to mediate between hypoglycemic and anti-osteoporosis effects. After insulin binds to the insulin receptor (IR), the PI3K/AKT signaling pathway is activated by phosphorylated insulin receptor substrates (IRS1&2) to achieve signal transduction. AKT phosphorylates and inhibits FOXO transcription function, thereby inactivating it. The four members of the FOXO family (FOXO1, 3, 4, and 6) are commonly expressed in mammals. FOXO1 is an essential transcriptional regulatory target molecule downstream of AKT. It regulates gluconeogenesis and glycogenolysis through insulin signaling [42], and its activation may lead to a high glucose state in the body. Moreover, FOXO1 is also a vital transcription factor that mediates RANKL-induced osteoclast formation. Experiments have proved that after knocking out the FOXO1 gene, the formation and activity of osteoclasts in vivo and in vitro can be reduced by half. At the same time, the proliferation of osteoblasts can be inhibited by inhibiting Wnt/β-catenin signal transduction [49]. The loss of FOXO1 in osteoprogenitor cells can also reduce the loss of cancellous bone mass in type 1 diabetes (T1DM) mice. There are still many relative reports on DOP, but this may be a treatment method to combat DOP bone mass loss. In conclusion, the PI3K/AKT/FOXO signaling pathway may be a key pathway in the pathogenesis of DOP, and LDP may play an active role in regulating blood sugar and improving bone quality.

#### VEGF signaling pathway

As we all know, the VEGF signaling pathway is the fundamental way to regulate angiogenesis [50]. However, VEGF receptors can also be expressed in many other types of cells, such as osteogenic precursor cells, osteoblasts, and osteoclasts. They can respond well to VEGF signaling [51], among which VEGFA is significantly better than other receptors in these cells in terms of function [52]. Through the VEGF signaling pathway, we can stimulate osteoblasts' bone formation and promote cartilage resorption and bone remodeling by osteoclasts. However, this homeostasis is disrupted in DM patients, indicating that anti-angiogenesis is a typical feature of their bones and bone marrow. In vitro studies have shown that a high-glucose environment may lead to the reduction in VEGF in osteoblasts [40], while in DM model mice, the arteries and capillaries of the bone marrow are sparse [53]. The perfusion rate of bone marrow is decreased, and the expression of VEGF in bone marrow cells is significantly reduced [54]. In vitro experiments have also shown that its ability to form blood vessels is weakened [55]. Relevant studies have shown that the application of VEGFA can prompt angiogenesis, bone formation, and bone reconstruction in the process of long bone regeneration in T2DM mice [41]. Therefore, inhibition of the VEGF signaling pathway may be one of the pathogenic mechanisms of DOP. The VEGF signaling pathway can also be coupled to the downstream PI3K/AKT pathway.

In conclusion, LDP may have an angiogenesis–osteogenesis coupling through the VEGF signal pathway and the VEGF/PI3K/AKT cascade, which will have a positive effect on DOP.

## Limitation

The results of this network construction are in line with our initial bioinformatics expectations: LDP can achieve the potential features of multiple targets–multiple channels treatment through the LDP–DOP network. This study demonstrates the two most effective herbs, five central compounds, seven core target genes, three biological processes, and two core pathways. But at the same time, we only pointed out that diosgenin has excellent research potential and value, and there is still a lack of in-depth excavation and research. In addition, although we found that the PI3K/AKT/FOXO and VEGF pathways may be the potential mechanisms of treatment, and these compounds can bind core proteins well in both pathways, we cannot ignore that the inference of these two pathways is based on the existing research on DOP. KEGG analysis also found some other potential enrichment pathways worthy of our further study.

In addition, this study is only used for the prediction and construction of network informatics, and its practicality and feasibility need to be further improved. It is necessary to carry out the clinical application and experimental implementation under conditions to judge the accuracy of this prediction. We sincerely hope that these findings can provide readers with some hints and new ideas.

## Conclusion

**Fig. 6:**
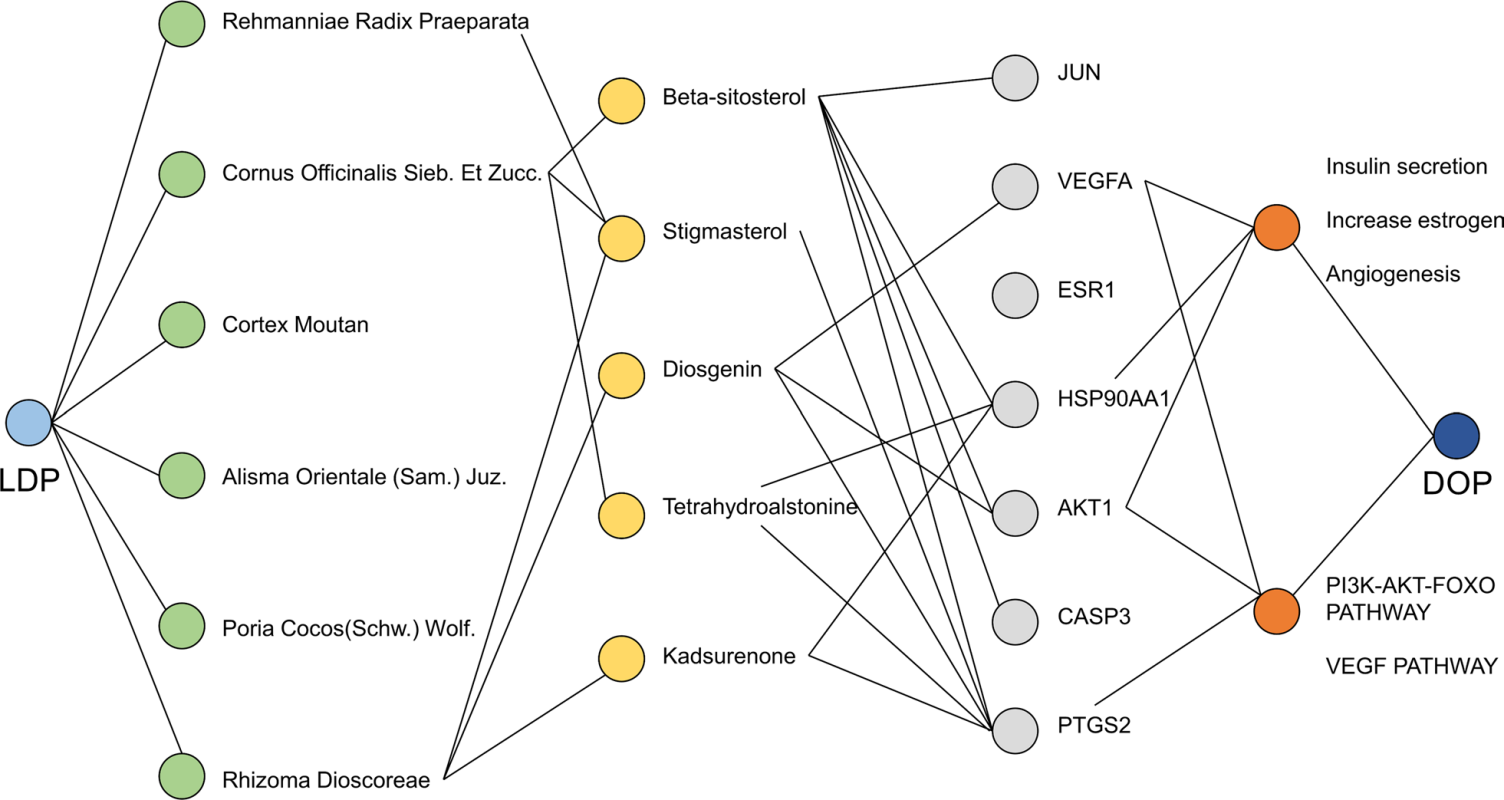
Relationship of LDP–herbs–compounds–targets–KEGG and GO-DOP

In conclusion, the use of network pharmacology and molecular docking in the study of LDP treatment in DOP reveals the potential pharmacological process and specific mechanisms that may be related to the several key active ingredients and signaling pathways (Fig. 6). Beta-sitosterol, stigmasterol, diosgenin, tetrahydroalstonine, and kadsurenone, the five most important compounds, may undergo the biological process of insulin secretion, thereby improving estrogen levels and promoting angiogenesis through key targets. At the same time, bioinformatics analysis confirms that PI3K/AKT/FOXO and VEGF signaling pathways may have higher reliability in DOP treatment. This new overall understanding is very consistent with our hypothesis and provides an opportunity for further research on the treatment of DOP and the exploitation of LDP. We hope that through this kind of bioinformatics prediction, we can get novel ideas and methods for clinical applications.

## Supplementary Information

**Additional file 1**.** Supplementary Table S1**. The significant GO entries enriched by the potential target genes.** Supplementary Table S2**. The significant KEGG pathway enriched by the potential target genes.

## Data Availability

The data and code used to support the findings of this study are available from the corresponding author upon reasonable request.

## Abbreviations

AGEs: Advanced glycation end products
BP: Biological process
Caco-2: Caco-2 permeability
CC: Cellular component
DL: Drug-likeness
DM: Diabetes mellitus
DOP: Diabetic osteoporosis
FDR: False discovery rate
FOXO: The forkhead box O
OC: Osteocalcin
OMIM: Online Mendelian Inheritance in Man
OPG: Osteoprotegerin
OS: Osteoporosis
PI3K: Phosphatidylinositol 3-kinase pathway
PPI: Protein–protein interaction
RANKL: Receptor activator of nuclear factor-κ B ligand
SERM: Selective estrogen receptor modulators
STRING: Search Tool for the Retrieval of Interacting Genes
STZ: Streptozotocin
T1DM: Type 1 diabetes mellitus
T2DM: Type 2 diabetes mellitus
TCMSP: Traditional Chinese Medicine Systems Pharmacology Database and Analysis Platform
UniProt: The Universal Protein Resource
VEGF: Vascular endothelial growth factor
VEGFA: Vascular endothelial growth factor A
