# Supplementary material for: Network pharmacology and molecular docking approach to elucidate the mechanisms of Liuwei Dihuang pill in diabetic osteoporosis
Source: J Orthop Surg Res. 2022 Jun 14;17:314. doi: 10.1186/s13018-022-03194-2 (PMC9195436; doi:10.1186/s13018-022-03194-2)
Supplement: Supplementary file 1 — Additional file 1. Supplementary Table S1. The significant GO entries enriched by the potential target genes. Supplementary Table S2. The significant KEGG pathway enriched by the potential target genes. [file 13018_2022_3194_MOESM1_ESM.docx]

**Supplementary Table S1**

| Category | Term | Count | PValue | Genes | FDR |
| --- | --- | --- | --- | --- | --- |
| biological process | response to estradiol | 6 | 1.74E-08 | CASP3, CASP9, PTGS2, CASP8, ESR1, CAT | 2.60E-05 |
| biological process | positive regulation of nitric oxide biosynthetic process | 5 | 6.63E-08 | AKT1, HSP90AA1, PTGS2, ESR1, MTOR | 9.89E-05 |
| biological process | response to drug | 7 | 2.30E-07 | CDKN1A, CASP3, HSP90AA1, PTGS2, RELA, JUN, CAT | 3.44E-04 |
| biological process | positive regulation of transcription from RNA polymerase II promoter | 9 | 1.11E-06 | PGR, AKT1, AR, RELA, JUN, GSK3B, VEGFA, ESR1, NR3C1 | 0.0016624 |
| biological process | negative regulation of apoptotic process | 7 | 2.43E-06 | AKT1, CDKN1A, CASP3, RELA, GSK3B, VEGFA, CAT | 0.0036323 |
| biological process | response to antibiotic | 4 | 3.46E-06 | CASP3, HSP90AA1, CASP9, CASP8 | 0.005164 |
| biological process | response to cobalt ion | 3 | 1.27E-05 | CASP3, CASP9, CASP8 | 0.019004 |
| biological process | response to lipopolysaccharide | 5 | 1.46E-05 | CASP3, CASP9, PTGS2, JUN, CASP8 | 0.0217292 |
| biological process | aging | 5 | 1.49E-05 | AKT1, CASP9, RELA, JUN, CAT | 0.0222562 |
| biological process | positive regulation of smooth muscle cell proliferation | 4 | 2.35E-05 | AKT1, PTGS2, JUN, MTOR | 0.0350478 |
| biological process | positive regulation of endothelial cell proliferation | 4 | 3.58E-05 | AKT1, JUN, VEGFA, MTOR | 0.0533768 |
| biological process | cellular response to hypoxia | 4 | 9.61E-05 | AKT1, PTGS2, VEGFA, MTOR | 0.1432334 |
| biological process | execution phase of apoptosis | 3 | 1.01E-04 | AKT1, CASP3, CASP8 | 0.1510958 |
| biological process | mammary gland alveolus development | 3 | 1.15E-04 | AR, VEGFA, ESR1 | 0.1711307 |
| biological process | response to tumor necrosis factor | 3 | 2.52E-04 | CASP3, PTGS2, CASP8 | 0.3754606 |
| biological process | negative regulation of gene expression | 4 | 2.75E-04 | PGR, AKT1, CDKN1A, ESR1 | 0.4098454 |
| biological process | transcription initiation from RNA polymerase II promoter | 4 | 3.73E-04 | PGR, AR, ESR1, NR3C1 | 0.5557164 |
| biological process | response to amino acid | 3 | 3.89E-04 | CASP3, RELA, MTOR | 0.5794729 |
| biological process | extrinsic apoptotic signaling pathway in absence of ligand | 3 | 4.69E-04 | CASP3, CASP9, GSK3B | 0.6975553 |
| biological process | signal transduction | 7 | 4.72E-04 | PGR, AKT1, AR, HSP90AA1, ESR1, NR3C1, MTOR | 0.7023522 |
| biological process | peptidyl-threonine phosphorylation | 3 | 5.87E-04 | AKT1, GSK3B, MTOR | 0.8714678 |
| biological process | positive regulation of neuron apoptotic process | 3 | 7.51E-04 | CASP3, CASP9, JUN | 1.1150133 |
| biological process | response to hydrogen peroxide | 3 | 0.001056129 | CASP3, JUN, CAT | 1.5640581 |
| biological process | positive regulation of fibroblast proliferation | 3 | 0.001183376 | CDKN1A, JUN, ESR1 | 1.7509548 |
| biological process | cellular response to organic cyclic compound | 3 | 0.001411001 | AKT1, CASP3, CASP8 | 2.0844575 |
| biological process | regulation of inflammatory response | 3 | 0.001606997 | PTGS2, RELA, ESR1 | 2.3707725 |
| biological process | response to insulin | 3 | 0.001815253 | RELA, CAT, MTOR | 2.6741412 |
| biological process | positive regulation of peptidyl-serine phosphorylation | 3 | 0.00197944 | AKT1, GSK3B, VEGFA | 2.9126937 |
| biological process | cellular response to mechanical stimulus | 3 | 0.002035684 | AKT1, PTGS2, CASP8 | 2.9942881 |
| biological process | regulation of cellular response to heat | 3 | 0.002268208 | HSP90AA1, GSK3B, MTOR | 3.3309315 |
| biological process | chemical synaptic transmission, postsynaptic | 2 | 0.004755668 | AKT1, GSK3B | 6.8646807 |
| biological process | glial cell apoptotic process | 2 | 0.004755668 | CASP3, CASP9 | 6.8646807 |
| biological process | negative regulation of NFAT protein import into nucleus | 2 | 0.004755668 | GSK3B, MTOR | 6.8646807 |
| biological process | negative regulation of cell proliferation | 4 | 0.005800307 | AR, CDKN1A, PTGS2, JUN | 8.3124732 |
| biological process | peptidyl-serine phosphorylation | 3 | 0.006161375 | AKT1, GSK3B, MTOR | 8.8079851 |
| biological process | positive regulation of protein phosphorylation | 3 | 0.006353877 | AKT1, VEGFA, MTOR | 9.0711426 |
| biological process | positive regulation of cell migration involved in sprouting angiogenesis | 2 | 0.006651992 | PTGS2, VEGFA | 9.4772802 |
| biological process | positive regulation of NF-kappaB transcription factor activity | 3 | 0.006947779 | AR, RELA, CAT | 9.8785722 |
| biological process | regulation of glycogen biosynthetic process | 2 | 0.007598884 | AKT1, MTOR | 10.756081 |
| biological process | activation of cysteine-type endopeptidase activity involved in apoptotic process by cytochrome c | 2 | 0.008544928 | CASP3, CASP9 | 12.01689 |
| biological process | negative regulation of cell size | 2 | 0.009490128 | AKT1, MTOR | 13.259961 |
| biological process | positive regulation of transcription from RNA polymerase III promoter | 2 | 0.010434482 | AR, MTOR | 14.485542 |
| biological process | positive regulation of lipid biosynthetic process | 2 | 0.011377993 | AKT1, MTOR | 15.693878 |
| biological process | protein autophosphorylation | 3 | 0.01139106 | AKT1, GSK3B, MTOR | 15.710501 |
| biological process | response to hypoxia | 3 | 0.01139106 | CASP3, VEGFA, CAT | 15.710501 |
| biological process | transcription from RNA polymerase II promoter | 4 | 0.011799541 | RELA, JUN, ESR1, NR3C1 | 16.228581 |
| biological process | positive regulation of transcription, DNA-templated | 4 | 0.011924451 | AR, RELA, JUN, ESR1 | 16.386412 |
| biological process | regulation of myelination | 2 | 0.01232066 | AKT1, MTOR | 16.88521 |
| biological process | cellular response to hepatocyte growth factor stimulus | 2 | 0.013262484 | RELA, GSK3B | 18.059777 |
| biological process | positive regulation of lamellipodium assembly | 2 | 0.015143607 | HSP90AA1, MTOR | 20.359552 |
| biological process | response to muscle stretch | 2 | 0.015143607 | RELA, JUN | 20.359552 |
| biological process | apoptotic process | 4 | 0.015446719 | CASP3, CASP9, CASP8, NR3C1 | 20.724439 |
| biological process | response to hyperoxia | 2 | 0.016082908 | CDKN1A, CAT | 21.485219 |
| biological process | response to fatty acid | 2 | 0.016082908 | PTGS2, CAT | 21.485219 |
| biological process | monocyte differentiation | 2 | 0.016082908 | JUN, VEGFA | 21.485219 |
| biological process | positive regulation of neuron death | 2 | 0.016082908 | GSK3B, MTOR | 21.485219 |
| biological process | cellular response to DNA damage stimulus | 3 | 0.016346439 | AKT1, CDKN1A, CASP9 | 21.798362 |
| biological process | macrophage differentiation | 2 | 0.017021368 | CASP8, VEGFA | 22.595043 |
| biological process | regulation of apoptotic process | 3 | 0.017096384 | CASP9, CASP8, ESR1 | 22.683121 |
| biological process | positive regulation of blood vessel endothelial cell migration | 2 | 0.017958989 | AKT1, VEGFA | 23.689243 |
| biological process | platelet formation | 2 | 0.017958989 | CASP3, CASP9 | 23.689243 |
| biological process | angiogenesis | 3 | 0.018639994 | PTGS2, JUN, VEGFA | 24.474906 |
| biological process | positive regulation of pri-miRNA transcription from RNA polymerase II promoter | 2 | 0.018895772 | RELA, JUN | 24.768039 |
| biological process | cellular component disassembly involved in execution phase of apoptosis | 2 | 0.019831717 | CASP3, CASP8 | 25.831648 |
| biological process | response to X-ray | 2 | 0.020766825 | CDKN1A, CASP3 | 26.880282 |
| biological process | positive regulation of nitric-oxide synthase activity | 2 | 0.020766825 | AKT1, ESR1 | 26.880282 |
| biological process | positive regulation of protein complex assembly | 2 | 0.020766825 | GSK3B, VEGFA | 26.880282 |
| biological process | cellular response to vascular endothelial growth factor stimulus | 2 | 0.021701096 | AKT1, VEGFA | 27.91415 |
| biological process | response to morphine | 2 | 0.021701096 | RELA, MTOR | 27.91415 |
| biological process | regulation of nitric-oxide synthase activity | 2 | 0.024498899 | AKT1, HSP90AA1 | 30.929223 |
| biological process | positive regulation of gene expression | 3 | 0.025198405 | AR, VEGFA, MTOR | 31.664443 |
| biological process | growth | 2 | 0.026359931 | VEGFA, MTOR | 32.869166 |
| biological process | spinal cord development | 2 | 0.028217635 | AKT1, MTOR | 34.754845 |
| biological process | germ cell development | 2 | 0.028217635 | AKT1, MTOR | 34.754845 |
| biological process | response to cocaine | 2 | 0.030072015 | HSP90AA1, MTOR | 36.587772 |
| biological process | positive regulation of vasoconstriction | 2 | 0.030072015 | AKT1, PTGS2 | 36.587772 |
| biological process | heart morphogenesis | 2 | 0.030072015 | VEGFA, MTOR | 36.587772 |
| biological process | negative regulation of autophagy | 2 | 0.033770827 | AKT1, MTOR | 40.101207 |
| biological process | response to cold | 2 | 0.033770827 | HSP90AA1, CASP8 | 40.101207 |
| biological process | positive regulation of cell differentiation | 2 | 0.034693462 | AR, JUN | 40.948842 |
| biological process | ERBB2 signaling pathway | 2 | 0.035615271 | AKT1, HSP90AA1 | 41.784533 |
| biological process | negative regulation of extrinsic apoptotic signaling pathway | 2 | 0.035615271 | AR, RELA | 41.784533 |
| biological process | negative regulation of I-kappaB kinase/NF-kappaB signaling | 2 | 0.037456413 | CASP8, ESR1 | 43.420745 |
| biological process | protein catabolic process | 2 | 0.038375748 | AKT1, MTOR | 44.221596 |
| biological process | cellular response to UV | 2 | 0.041128816 | CASP9, PTGS2 | 46.557046 |
| biological process | outflow tract morphogenesis | 2 | 0.042960087 | JUN, VEGFA | 48.059652 |
| biological process | response to cAMP | 2 | 0.042960087 | RELA, JUN | 48.059652 |
| biological process | positive regulation of cell division | 2 | 0.043874493 | VEGFA, CAT | 48.795103 |
| biological process | response to heat | 2 | 0.044788079 | AKT1, HSP90AA1 | 49.520183 |
| biological process | inflammatory response | 3 | 0.049494722 | AKT1, PTGS2, RELA | 53.106292 |
| molecular function | enzyme binding | 7 | 3.82E-07 | PGR, AKT1, AR, PTGS2, JUN, ESR1, CAT | 4.53E-04 |
| molecular function | transcriptional activator activity, RNA polymerase II core promoter proximal region sequence-specific binding | 6 | 1.97E-06 | PGR, AR, RELA, JUN, ESR1, NR3C1 | 0.0023408 |
| molecular function | steroid binding | 4 | 2.02E-06 | PGR, AR, ESR1, NR3C1 | 0.0023924 |
| molecular function | RNA polymerase II core promoter proximal region sequence-specific DNA binding | 6 | 1.44E-05 | PGR, AR, RELA, JUN, ESR1, NR3C1 | 0.0171311 |
| molecular function | steroid hormone receptor activity | 4 | 1.88E-05 | PGR, AR, ESR1, NR3C1 | 0.0222934 |
| molecular function | nitric-oxide synthase regulator activity | 3 | 2.35E-05 | AKT1, HSP90AA1, ESR1 | 0.0279004 |
| molecular function | protein complex binding | 5 | 3.49E-05 | CDKN1A, CASP3, RELA, CASP8, NR3C1 | 0.0414729 |
| molecular function | identical protein binding | 7 | 4.09E-05 | AKT1, HSP90AA1, RELA, JUN, CASP8, VEGFA, ESR1 | 0.0484876 |
| molecular function | cysteine-type endopeptidase activity involved in apoptotic process | 3 | 6.53E-05 | CASP3, CASP9, CASP8 | 0.0774904 |
| molecular function | protein homodimerization activity | 6 | 4.37E-04 | HSP90AA1, PTGS2, RELA, JUN, VEGFA, CAT | 0.518123 |
| molecular function | protein binding | 16 | 4.53E-04 | AR, HSP90AA1, PTGS2, RELA, ESR1, NR3C1, AKT1, PGR, CDKN1A, CASP3, CASP9, GSK3B, JUN, CASP8, VEGFA, MTOR | 0.5369456 |
| molecular function | sequence-specific DNA binding | 5 | 0.001188734 | PGR, AR, JUN, ESR1, NR3C1 | 1.4021341 |
| molecular function | kinase activity | 4 | 0.001402531 | AKT1, CDKN1A, GSK3B, MTOR | 1.6523927 |
| molecular function | cysteine-type endopeptidase activity | 3 | 0.001491865 | CASP3, CASP9, CASP8 | 1.7567894 |
| molecular function | transcription factor activity, sequence-specific DNA binding | 6 | 0.001525071 | PGR, AR, RELA, JUN, ESR1, NR3C1 | 1.7955688 |
| molecular function | ATPase binding | 3 | 0.002186177 | PGR, AR, ESR1 | 2.56472 |
| molecular function | transcription factor binding | 4 | 0.002242993 | AR, RELA, JUN, ESR1 | 2.6305633 |
| molecular function | ubiquitin protein ligase binding | 4 | 0.002311067 | CDKN1A, RELA, GSK3B, CASP8 | 2.7093988 |
| molecular function | beta-catenin binding | 3 | 0.002676158 | AR, GSK3B, ESR1 | 3.1312141 |
| molecular function | peptidase activity | 3 | 0.003213126 | CASP3, CASP9, CASP8 | 3.7485685 |
| molecular function | protein kinase binding | 4 | 0.004947917 | CASP9, RELA, GSK3B, MTOR | 5.718525 |
| molecular function | chromatin binding | 4 | 0.005517576 | AR, RELA, JUN, ESR1 | 6.3573086 |
| molecular function | death receptor binding | 2 | 0.015064299 | CASP3, CASP8 | 16.489512 |
| molecular function | DNA binding | 6 | 0.01638834 | PGR, AR, RELA, JUN, ESR1, NR3C1 | 17.812551 |
| molecular function | transcription regulatory region DNA binding | 3 | 0.016926995 | AR, RELA, JUN | 18.345283 |
| molecular function | NF-kappaB binding | 2 | 0.028070778 | RELA, GSK3B | 28.681293 |
| molecular function | RNA polymerase II transcription factor activity, ligand-activated sequence-specific DNA binding | 2 | 0.033595541 | AR, ESR1 | 33.348062 |
| molecular function | receptor binding | 3 | 0.043133071 | PGR, AR, CAT | 40.751507 |
| molecular function | RNA polymerase II transcription factor binding | 2 | 0.043647885 | AR, GSK3B | 41.128832 |
| molecular function | protein kinase activity | 3 | 0.044467579 | AKT1, GSK3B, MTOR | 41.725075 |
| molecular function | protein serine/threonine kinase activity | 3 | 0.048332965 | AKT1, GSK3B, MTOR | 44.462931 |
| cell composition | nucleoplasm | 12 | 2.15E-06 | PGR, AKT1, AR, CDKN1A, CASP3, HSP90AA1, RELA, JUN, CASP8, ESR1, NR3C1, MTOR | 0.0023089 |
| cell composition | cytosol | 12 | 1.26E-05 | AKT1, AR, CDKN1A, CASP3, HSP90AA1, CASP9, RELA, JUN, GSK3B, CASP8, CAT, MTOR | 0.0135089 |
| cell composition | protein complex | 6 | 2.05E-05 | AKT1, AR, CDKN1A, HSP90AA1, PTGS2, NR3C1 | 0.0220486 |
| cell composition | nucleus | 14 | 2.97E-05 | AR, HSP90AA1, PTGS2, RELA, ESR1, NR3C1, AKT1, PGR, CASP3, CDKN1A, CASP9, GSK3B, JUN, MTOR | 0.0319936 |
| cell composition | cytoplasm | 12 | 0.001022251 | AKT1, AR, CASP3, HSP90AA1, PTGS2, RELA, GSK3B, CASP8, VEGFA, ESR1, NR3C1, MTOR | 1.0943424 |
| cell composition | death-inducing signaling complex | 2 | 0.006130585 | CASP3, CASP8 | 6.4019197 |
| cell composition | mitochondrial outer membrane | 3 | 0.007391187 | PGR, CASP8, MTOR | 7.6712963 |
| cell composition | nuclear chromatin | 3 | 0.012144359 | AR, RELA, ESR1 | 12.318366 |
| cell composition | neuron projection | 3 | 0.017924103 | HSP90AA1, PTGS2, CASP8 | 17.68277 |
| cell composition | postsynapse | 2 | 0.019149101 | AKT1, GSK3B | 18.780766 |
| cell composition | mitochondrion | 5 | 0.025364491 | AKT1, CASP9, GSK3B, CASP8, CAT | 24.149828 |
| cell composition | neuronal cell body | 3 | 0.030463591 | HSP90AA1, GSK3B, MTOR | 28.311906 |

**Supplementary Table S2**

| Category | Term | Count | PValue | Genes | FDR |
| --- | --- | --- | --- | --- | --- |
| kegg pathway | Pathways in cancer | 13 | 1.52E-12 | AR, HSP90AA1, PTGS2, RELA, AKT1, CDKN1A, CASP3, CASP9, GSK3B, JUN, CASP8, VEGFA, MTOR | 1.74E-09 |
| kegg pathway | Prostate cancer | 8 | 4.59E-10 | AKT1, AR, CDKN1A, HSP90AA1, CASP9, RELA, GSK3B, MTOR | 5.25E-07 |
| kegg pathway | Hepatitis B | 7 | 5.32E-07 | AKT1, CDKN1A, CASP3, CASP9, RELA, JUN, CASP8 | 6.09E-04 |
| kegg pathway | TNF signaling pathway | 6 | 3.16E-06 | AKT1, CASP3, PTGS2, RELA, JUN, CASP8 | 0.0036162 |
| kegg pathway | PI3K-Akt signaling pathway | 8 | 5.80E-06 | AKT1, CDKN1A, HSP90AA1, CASP9, RELA, GSK3B, VEGFA, MTOR | 0.0066415 |
| kegg pathway | Apoptosis | 5 | 1.00E-05 | AKT1, CASP3, CASP9, RELA, CASP8 | 0.0114917 |
| kegg pathway | Colorectal cancer | 5 | 1.00E-05 | AKT1, CASP3, CASP9, JUN, GSK3B | 0.0114917 |
| kegg pathway | Non-alcoholic fatty liver disease (NAFLD) | 6 | 1.71E-05 | AKT1, CASP3, RELA, JUN, GSK3B, CASP8 | 0.0196125 |
| kegg pathway | ErbB signaling pathway | 5 | 3.87E-05 | AKT1, CDKN1A, JUN, GSK3B, MTOR | 0.0442662 |
| kegg pathway | HIF-1 signaling pathway | 5 | 5.70E-05 | AKT1, CDKN1A, RELA, VEGFA, MTOR | 0.0652272 |
| kegg pathway | Proteoglycans in cancer | 6 | 6.65E-05 | AKT1, CDKN1A, CASP3, VEGFA, ESR1, MTOR | 0.0760406 |
| kegg pathway | Toxoplasmosis | 5 | 9.71E-05 | AKT1, CASP3, CASP9, RELA, CASP8 | 0.1111248 |
| kegg pathway | Thyroid hormone signaling pathway | 5 | 1.16E-04 | AKT1, CASP9, GSK3B, ESR1, MTOR | 0.1321258 |
| kegg pathway | Legionellosis | 4 | 2.38E-04 | CASP3, CASP9, RELA, CASP8 | 0.2723211 |
| kegg pathway | VEGF signaling pathway | 4 | 3.42E-04 | AKT1, CASP9, PTGS2, VEGFA | 0.3910093 |
| kegg pathway | Pancreatic cancer | 4 | 4.13E-04 | AKT1, CASP9, RELA, VEGFA | 0.471693 |
| kegg pathway | p53 signaling pathway | 4 | 4.52E-04 | CDKN1A, CASP3, CASP9, CASP8 | 0.5157461 |
| kegg pathway | B cell receptor signaling pathway | 4 | 4.93E-04 | AKT1, RELA, JUN, GSK3B | 0.562354 |
| kegg pathway | Prolactin signaling pathway | 4 | 5.36E-04 | AKT1, RELA, GSK3B, ESR1 | 0.6115759 |
| kegg pathway | Influenza A | 5 | 5.67E-04 | AKT1, CASP9, RELA, JUN, GSK3B | 0.6472512 |
| kegg pathway | Tuberculosis | 5 | 6.05E-04 | AKT1, CASP3, CASP9, RELA, CASP8 | 0.6903999 |
| kegg pathway | Small cell lung cancer | 4 | 9.08E-04 | AKT1, CASP9, PTGS2, RELA | 1.0340419 |
| kegg pathway | Viral carcinogenesis | 5 | 0.0010516 | CDKN1A, CASP3, RELA, JUN, CASP8 | 1.1969151 |
| kegg pathway | Estrogen signaling pathway | 4 | 0.0014132 | AKT1, HSP90AA1, JUN, ESR1 | 1.6054908 |
| kegg pathway | T cell receptor signaling pathway | 4 | 0.0014549 | AKT1, RELA, JUN, GSK3B | 1.6524351 |
| kegg pathway | Chagas disease (American trypanosomiasis) | 4 | 0.0016292 | AKT1, RELA, JUN, CASP8 | 1.8487137 |
| kegg pathway | Toll-like receptor signaling pathway | 4 | 0.001721 | AKT1, RELA, JUN, CASP8 | 1.9520215 |
| kegg pathway | Insulin resistance | 4 | 0.0018161 | AKT1, RELA, GSK3B, MTOR | 2.0588249 |
| kegg pathway | HTLV-I infection | 5 | 0.0023255 | AKT1, CDKN1A, RELA, JUN, GSK3B | 2.6292931 |
| kegg pathway | Neurotrophin signaling pathway | 4 | 0.002456 | AKT1, RELA, JUN, GSK3B | 2.7749693 |
| kegg pathway | Epstein-Barr virus infection | 4 | 0.0025746 | AKT1, CDKN1A, RELA, JUN | 2.9071739 |
| kegg pathway | Hepatitis C | 4 | 0.0032907 | AKT1, CDKN1A, RELA, GSK3B | 3.701933 |
| kegg pathway | MicroRNAs in cancer | 5 | 0.0035816 | CDKN1A, CASP3, PTGS2, VEGFA, MTOR | 4.0230982 |
| kegg pathway | Amyotrophic lateral sclerosis (ALS) | 3 | 0.0058222 | CASP3, CASP9, CAT | 6.4642721 |
| kegg pathway | Endometrial cancer | 3 | 0.0062852 | AKT1, CASP9, GSK3B | 6.9615816 |
| kegg pathway | Alzheimer's disease | 4 | 0.0063401 | CASP3, CASP9, GSK3B, CASP8 | 7.020398 |
| kegg pathway | NOD-like receptor signaling pathway | 3 | 0.0072602 | HSP90AA1, RELA, CASP8 | 8.0008982 |
| kegg pathway | Acute myeloid leukemia | 3 | 0.0072602 | AKT1, RELA, MTOR | 8.0008982 |
| kegg pathway | Viral myocarditis | 3 | 0.007514 | CASP3, CASP9, CASP8 | 8.2697444 |
| kegg pathway | Herpes simplex infection | 4 | 0.0080333 | CASP3, RELA, JUN, CASP8 | 8.8175665 |
| kegg pathway | Glioma | 3 | 0.0096873 | AKT1, CDKN1A, MTOR | 10.542391 |
| kegg pathway | Renal cell carcinoma | 3 | 0.0099765 | AKT1, JUN, VEGFA | 10.840935 |
| kegg pathway | Epithelial cell signaling in Helicobacter pylori infection | 3 | 0.0102696 | CASP3, RELA, JUN | 11.142522 |
| kegg pathway | Focal adhesion | 4 | 0.0111104 | AKT1, JUN, GSK3B, VEGFA | 12.002628 |
| kegg pathway | Adipocytokine signaling pathway | 3 | 0.0111717 | AKT1, RELA, MTOR | 12.065021 |
| kegg pathway | Leishmaniasis | 3 | 0.01148 | PTGS2, RELA, JUN | 12.37826 |
| kegg pathway | Chronic myeloid leukemia | 3 | 0.011792 | AKT1, CDKN1A, RELA | 12.694284 |
| kegg pathway | Pertussis | 3 | 0.0127505 | CASP3, RELA, JUN | 13.658546 |
| kegg pathway | Progesterone-mediated oocyte maturation | 3 | 0.0169127 | PGR, AKT1, HSP90AA1 | 17.733841 |
| kegg pathway | MAPK signaling pathway | 4 | 0.0193024 | AKT1, CASP3, RELA, JUN | 19.99366 |
| kegg pathway | Choline metabolism in cancer | 3 | 0.0224031 | AKT1, JUN, MTOR | 22.841242 |
| kegg pathway | Osteoclast differentiation | 3 | 0.0362779 | AKT1, RELA, JUN | 34.485313 |
| kegg pathway | Measles | 3 | 0.0372978 | AKT1, RELA, GSK3B | 35.274464 |
| kegg pathway | FoxO signaling pathway | 3 | 0.037812 | AKT1, CDKN1A, CAT | 35.669002 |
| kegg pathway | Insulin signaling pathway | 3 | 0.0398965 | AKT1, GSK3B, MTOR | 37.24608 |
| kegg pathway | Oxytocin signaling pathway | 3 | 0.0464092 | CDKN1A, PTGS2, JUN | 41.948772 |
